# Isolation and characterization of camelid single-domain antibodies against HER2

**DOI:** 10.1186/s13104-018-3955-8

**Published:** 2018-12-05

**Authors:** Greg Hussack, Shalini Raphael, Michael J. Lowden, Kevin A. Henry

**Affiliations:** Human Health Therapeutics Research Centre, National Research Council Canada, 100 Sussex Drive, Ottawa, ON K1A 0R6 Canada

**Keywords:** Single-domain antibody, V_H_H, HER2, Cancer

## Abstract

**Objective:**

To isolate and characterize novel high-affinity llama single-domain antibodies against human HER2.

**Results:**

We immunized a llama with human HER2, constructed a phage-displayed V_H_H library from the lymphocytes of the animal, and isolated six unique HER2-specific V_H_Hs by panning. All six V_H_Hs were unique at the amino acid level and were clonally unrelated, as reflected by their distinct CDR3 lengths. All six V_H_Hs recognized recombinant human HER2 ectodomain with monovalent affinities ranging from 1 to 51 nM, had comparable affinities for cynomolgus monkey HER2, and bound HER2^+^ SKOV3 cells by flow cytometry. Three of the V_H_Hs recognized recombinant murine HER2 with no loss of affinity compared with human and cynomolgus monkey HER2. The V_H_Hs recognized three major epitopes on HER2 (including one conserved across the human, simian and murine orthologues), all of which were distinct from that of trastuzumab. These V_H_Hs may be useful in the design of modular cancer immunotherapeutics.

**Electronic supplementary material:**

The online version of this article (10.1186/s13104-018-3955-8) contains supplementary material, which is available to authorized users.

## Introduction

HER2/neu (ERBB2, CD340) is a receptor tyrosine kinase of the epidermal growth factor receptor family that is frequently amplified and/or overexpressed in solid tumors [1]. Antibodies (Abs) and Ab-drug conjugates (ADCs) against HER2, exemplified by trastuzumab (Herceptin), pertuzumab (Perjeta) and trastuzumab emtansine (Kadcyla), play an important role in the diagnosis and treatment of breast cancer [2]. Multiple other anti-HER2 Abs and Ab fragments are in development as naked antibodies, ADCs, bispecific Abs and radioimmunotherapeutics for breast cancer and other indications [3]. A camelid single-domain Ab (sdAb or V_H_H) against HER2 is currently in clinical trials for breast cancer imaging [4].

Here, we report the generation and preliminary characterization of a panel of novel llama V_H_Hs directed against human HER2. Several of the V_H_Hs have attractive properties that may make them useful components of modular cancer immunotherapeutics.

## Main text

We immunized a male llama (*Lama glama*) with recombinant human HER2 ectodomain (Cat. No. HE2-H5225; ACROBiosystems, Beijing, China) as previously described [5–7]. Briefly, the animal was immunized subcutaneously five times with 200 µg of human HER2 (days 0, 21, 28, 35 and 42). The priming immunization was adjuvanted with complete Freund’s adjuvant and boost immunizations were adjuvanted with incomplete Freund’s adjuvant. Blood samples were collected on days 35 and 49, from which serum was obtained after clotting and peripheral blood mononuclear cells were purified by density gradient centrifugation. Interestingly, serum ELISA and western blotting indicated that although HER2 immunization elicited polyclonal Abs against the immunizing antigen, immune sera from unrelated animals showed similar degrees of HER2 reactivity (see Additional file 1). We speculate that serum polyreactivity against this recombinant HER2 ectodomain reflects some degree of unfolding and/or aggregation, although binding by trastuzumab and other antibodies indicated that some proportion was also folded correctly.

We constructed a phage-displayed V_H_H library from the peripheral blood lymphocytes of the HER2-immunized llama as previously described [5–7]. Briefly, total RNA was extracted in 16 replicates from peripheral blood mononuclear cells (eight samples each from the day 35 and 49 bleeds, each containing ~ 1 × 10^7^ cells) using the PureLink™ RNA Mini Kit (Thermo Fisher, Waltham, MA). Approximately 1–2 µg of total RNA was reverse transcribed using qScript^®^ cDNA SuperMix (Quantabio, Beverly, MA) and then rearranged V_H_H exons were amplified using semi-nested PCR and cloned into the pMED1 phagemid vector. The final library size was ~ 8 × 10^7^ independent transformants with an insert rate of ~ 92%. V_H_H-displaying phages were rescued from library phagemid-bearing *Escherichia coli* TG1 cells using M13KO7 helper phage (New England Biolabs, Ipswich, MA), panned for four rounds against human HER2 directly immobilized in wells of microtiter plates, and eluted with triethylamine as previously described [5–7]. At the conclusion of four rounds of panning, 96 individual clones (48 each from rounds 3 and 4) were tested for binding to human HER2 by ELISA, yielding six unique and clonally unrelated V_H_H sequences (Table 1).

**Table 1:** Properties of HER2-specific V_H_Hs isolated in this study

V_H_H	CDR3 Length (aa)^a^	Human HER2			Cynomolgus HER2			Mouse HER2		
		k_on_ (M^−1^s^−1^)	k_off_ (s^−1^)	*K*_*D*_ (nM)	k_on_ (M^−1^s^−1^)	k_off_ (s^−1^)	*K*_*D*_ (nM)	k_on_ (M^−1^s^−1^)	k_off_ (s^−1^)	*K*_*D*_ (nM)
NRC-sdAb034	12	5.4 × 10^5^	3.4 × 10^−3^	6.2	5.8 × 10^5^	2.7 × 10^−3^	4.6	5.0 × 10^5^	2.9 × 10^−3^	5.8
NRC-sdAb035	15	8.5 × 10^5^	4.4 × 10^−2^	51	1.0 × 10^6^	4.3 × 10^−2^	41			n.b.
NRC-sdAb036	19	3.9 × 10^5^	5.6 × 10^−3^	14	4.8 × 10^5^	5.4 × 10^−3^	11	4.4 × 10^5^	5.8 × 10^−3^	13
NRC-sdAb037	16	4.3 × 10^5^	1.7 × 10^−2^	39	5.1 × 10^5^	5.6 × 10^−2^	110			n.b.
NRC-sdAb038	10	1.1 × 10^6^	1.6 × 10^−3^	1.4	1.0 × 10^6^	1.4 × 10^−3^	1.3	1.1 × 10^6^	1.0 × 10^−3^	0.9
NRC-sdAb039	18	1.4 × 10^6^	4.5 × 10^−3^	3.3	1.7 × 10^6^	6.0 × 10^−3^	3.6			n.b.

*n.b.* no binding

^a^IMGT numbering

**Fig. 1:**
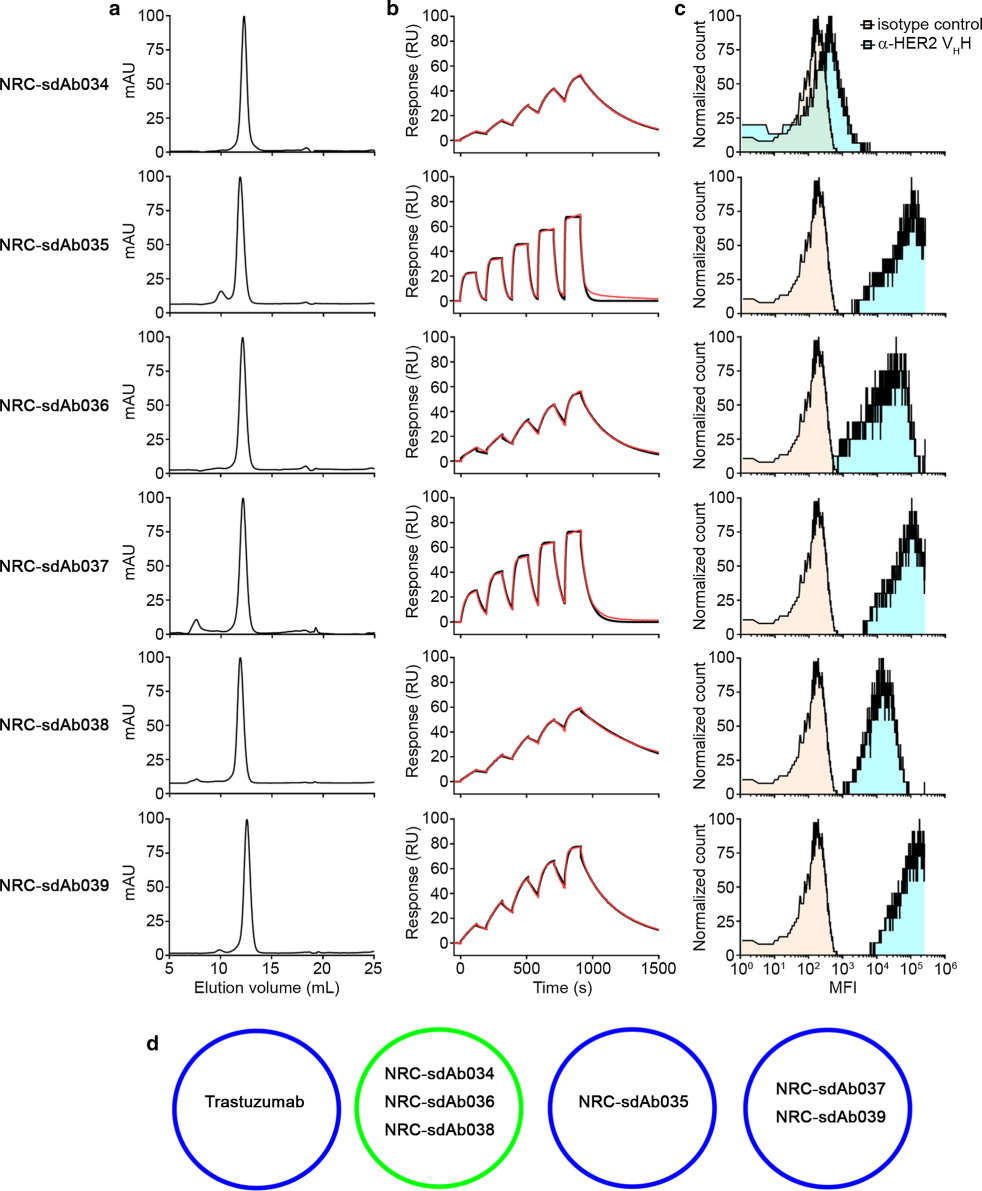
Characterization of anti-HER2 llama V_H_Hs. **a** Size exclusion chromatography profiles of anti-HER2 V_H_Hs. Approximately 0.5 mg of each V_H_H was injected over a Superdex™ 75 GL column (GE Healthcare) connected to an ÄKTA FPLC protein purification system (GE Healthcare) in a mobile phase consisting of HBS-EP + (10 mM HEPES, pH 7.4, containing 150 mM NaCl, 3 mM EDTA and 0.05% surfactant P20). Maximum A_280_ values were normalized to 100 for each V_H_H. **b** Single-cycle kinetic analysis of V_H_Hs binding to human HER2 by SPR. All V_H_Hs were purified by preparative size exclusion chromatography prior to analysis. Approximately 1323 response units (RUs) of human HER2 were immobilized on adjacent flow cells of a CM5 Series S sensor chip in 10 mM acetate, pH 4.0, using an amine coupling kit (GE Healthcare). An ethanolamine-blocked flow cell served as the reference. Monomeric V_H_Hs at concentrations ranging from 1–400 nM were injected over the surfaces in HBS-EP+ buffer at a flow rate of 40 µL min^−1^. The contact time was 120 s and the dissociation time was 600 s. The surfaces were regenerated using 10 mM glycine, pH 1.5. Data were analyzed using Biacore T200 Software v3.0 (GE Healthcare) and fitted to a 1:1 binding model (black lines show data and red lines show fits). Affinity and kinetic parameters (25 °C) are shown in Table 1, and sensorgrams showing binding to cynomolgus and murine HER2 are shown in Additional file 2. **c** Binding of V_H_Hs to HER2^+^ SKOV3 cells by flow cytometry. SKOV3 cells were grown to 70–80% confluency at 37 °C in a humidified 5% CO_2_ atmosphere in RPMI-1640 medium supplemented with 10% fetal bovine serum, 100 U mL^−1^ penicillin, 100 µg mL^−1^ streptomycin and 250 ng mL^−1^ amphotericin B. Cells were dissociated from flasks using Accutase^®^ solution, washed in PBS and then resuspended in PBS containing 1% bovine serum albumin. Approximately 1 × 10^5^ cells were stained sequentially on ice for 30 min with: (i) 10 µg mL^−1^ of each V_H_H, (ii) 5 µg mL^−1^ of mouse anti-c-Myc IgG (clone 9E10), and (iii) 5 µg mL^−1^ of APC-conjugated goat anti-mouse IgG (Thermo-Fisher). The cells were washed with PBS in between each staining step, and after the final wash, data (10,000 events) were acquired on a BD FACSCanto™ instrument (BD Biosciences, San Jose, CA). **d** Summary of epitope binning of anti-HER2 V_H_Hs by SPR. HER2 was immobilized as described in B. In the first injection, each V_H_H at a concentration equivalent to 20 × *K*_*D*_ or trastuzumab (20 nM) was injected at a flow rate of 20 µL s^−1^ for 300 s contact time to saturate the HER2 surface. The second injection consisted of the same V_H_H along with a second V_H_H (both at 20 × their respective *K*_*D*_s). All co injection experiments were performed in both orientations. Blue circles represent distinct epitopes conserved between human and cynomolgus HER2, and green circle represents a distinct epitope conserved across human, cynomolgus and mouse HER2

The DNA sequences encoding the six V_H_Hs were cloned into the pSJF2H expression vector [8]. C-terminally c-Myc- and His_6_-tagged V_H_Hs were expressed in 200 mL overnight cultures of *E. coli* TG1 under IPTG induction and purified by Ni^2+^ affinity chromatography as previously described [5–7]. All six V_H_Hs were primarily monomeric by size exclusion chromatography, although trace aggregates or impurities were observed for NRC-sdAb035 and NRC-sdAb037 (Fig. 1a). We immobilized human (ACROBiosystems HE2-H5225), cynomolgus (ACROBiosystems HE2-C52Hb) and murine HER2 (ACROBiosystems ER2-M5220) ectodomains on adjacent flow cells of a CM5 Series S sensor chip (GE Healthcare, Piscataway, NJ) by amine coupling and analyzed binding of the V_H_Hs to each surface using single-cycle kinetics on a Biacore T200 surface plasmon resonance (SPR) instrument (GE Healthcare). All six V_H_Hs showed high-affinity binding to HER2 (*K*_*D*_ range 1–51 nM), with nearly equivalent kinetic and affinity parameters observed for human and cynomolgus HER2 (Fig. 1b, Table 1 and Additional file 2); moreover, three of the V_H_Hs also cross-reacted with murine HER2 with no apparent loss of binding affinity. All six V_H_Hs bound to HER2^+^ SKOV3 cells by flow cytometry, although staining by NRC-sdAb034 was weak (Fig. 1c). Epitope binning experiments indicated that despite their unique amino acid sequences, all three cross-reactive V_H_Hs (NRC-sdAb034, NRC-sdAb036 and NRC-sdAb038) targeted a nearly identical epitope (Fig. 1d and Additional file 3); the epitopes of NRC-sdAb037 and NRC-sdAb039 also showed a high degree of overlap, while NRC-sdAb035’s epitope was distinct. The epitopes of all six V_H_Hs were distinct from the trastuzumab epitope.

In summary, we have reported the isolation and preliminary characterization of six novel anti-HER2 llama V_H_Hs. Interestingly, despite their unique and clonally unrelated sequences, the V_H_Hs targeted only three major epitopes on HER2, including an apparently immunodominant epitope conserved across the human, simian and murine orthologues. The nearly identical binding of the V_H_Hs to cynomolgus HER2 would permit toxicity assessment and, for mouse cross-reactive V_H_Hs, evaluation in syngeneic tumor models in combination with immunomodulatory agents. Several other groups have described anti-HER2 V_H_Hs and characterized them in some detail [9], but in most cases cross-reactivity with murine HER2 was not assessed. Moreover, targeting of a non-trastuzumab epitope is clearly an advantage for imaging of patients being dosed with Herceptin/Kadcyla [10]. The other major potential advantage of these V_H_Hs in comparison with conventional antibodies is their modularity, which would permit facile incorporation into multifunctional biologics.

## Limitations

At the current time, we have not been able to explore whether these V_H_Hs have anti-cancer activity. We do not know: (i) the precise locations of their epitopes on HER2, (ii) whether any of the V_H_Hs inhibit HER2 signaling or inhibit receptor dimerization, permitting their development as naked Ab therapeutics, (iii) whether any of the V_H_Hs internalize into HER2^+^ tumor cells, permitting their development as ADCs or radioimmunotherapeutics, or (iv) whether their sequences can be humanized without loss of stability or binding affinity. Moreover, we are unable to disclose the amino acid sequences of these V_H_Hs for intellectual property reasons. In future studies, we hope to comprehensively investigate the roles of molecular size, valency and serum half-life on tumor uptake using anti-HER2 V_H_H-based biologics as a model system.

## Additional files

**Additional file 1: Figure S1.** Polyclonal antibody responses after immunization with human HER2 ectodomain.

**Additional file 2: Figure S2.** Complete sensorgrams for single-cycle kinetic analysis of V_H_Hs binding to HER2 by SPR.

**Additional file 3: Figure S3.** Complete sensorgrams for epitope binning SPR co-injection experiments.

## Abbreviations

Ab: antibody
ADC: antibody-drug conjugate
sdAb or V_H_H: single-domain antibody
SPR: surface plasmon resonance
