# Supplementary material for: Isolation and characterization of camelid single-domain antibodies against HER2
Source: BMC Res Notes. 2018 Dec 5;11:866. doi: 10.1186/s13104-018-3955-8 (PMC6282393; doi:10.1186/s13104-018-3955-8)
Supplement: Supplementary file 1 — Additional file 1: Figure S1. Polyclonal antibody responses after immunization with human HER2 ectodomain. [file 13104_2018_3955_MOESM1_ESM.pdf]

A

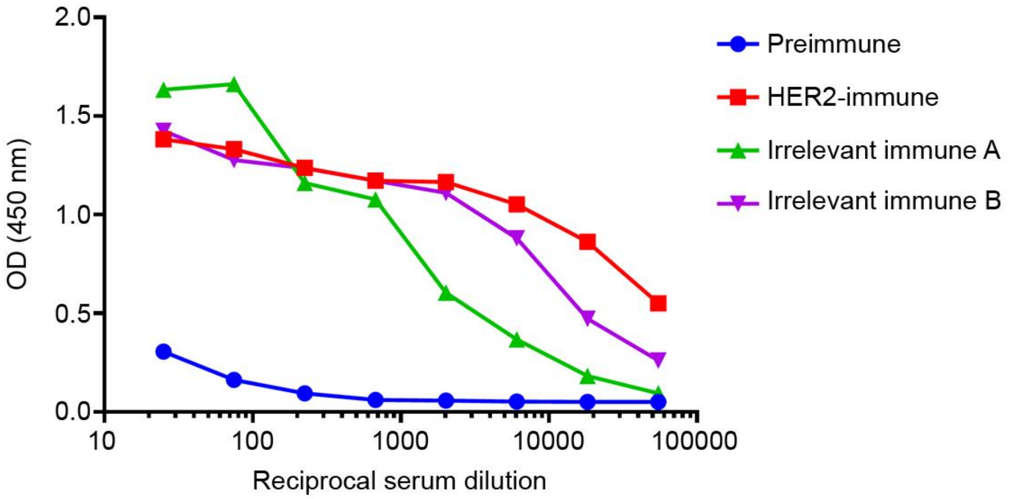

B

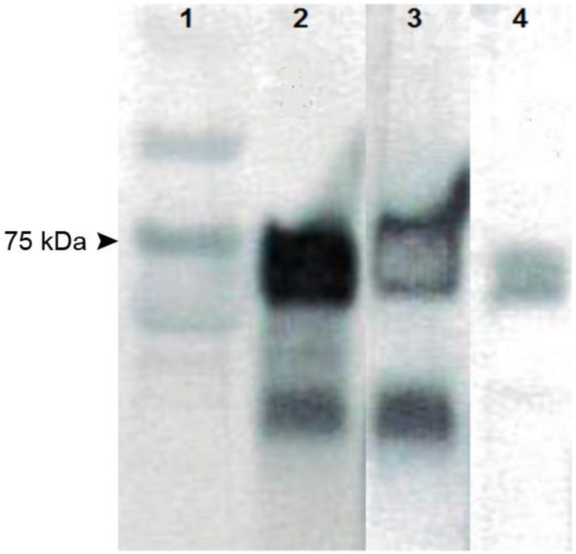

Figure S1. Polyclonal antibody responses after immunization with human HER2 ectodomain. **(A)** Titration serum ELISA against human HER2. Wells of microtiter plates were coated overnight at 4°C with 100 ng of human HER2 (Acro HE2-H5225) in 35 µL of PBS. The next day, wells were blocked with 200 µL of PBS containing 2% (w/v) dry milk for 1 h at 37°C, then 35 µL of serum (serially diluted in PBS containing 1% dry milk and 0.1% Tween-20) were added to wells for 2 h at room temperature. After five washes with PBS containing 0.1% Tween-20, HRP-conjugated polyclonal goat anti-llama IgG (1/5000, Cedarlane Labs A160-100P) was added for 45 min at room temperature. Wells were developed with TMB substrate, stopped with 1 M H_2SO_4 and the absorbance at 450 nm was measured. Sera from two llamas (A and B) immunized with irrelevant His-tagged proteins with no homology to HER2 are shown for reference. **(B)** Western blotting of human HER2 (Acro HE2-H5525) with immune llama serum. Approximately 1 µg of HER2 was electrophoresed in Mini-PROTEAN® TGX 4-20% stain-free gels (Bio-Rad) and transferred to PVDF membranes. After blocking with 5% dry milk overnight, the membranes were blotted with llama serum (1/1000) diluted in PBS containing 2% dry milk and 0.1% Tween-20 for 2 h, then with HRP-conjugated goat anti-llama IgG (1/5000) for 1 h and developed with Pierce ECL substrate. Molecular weight marker is Bio-Rad Precision Plus Protein™ Unstained Protein Standards, Cat. #1610363. Lane 1, molecular weight standards, lane 2, HER2-immunized llama, lane 3, llama B immunized with irrelevant His-tagged proteins, lane 4, llama C immunized with irrelevant His-tagged proteins.
