# Supplementary material for: Isolation and characterization of camelid single-domain antibodies against HER2
Source: BMC Res Notes. 2018 Dec 5;11:866. doi: 10.1186/s13104-018-3955-8 (PMC6282393; doi:10.1186/s13104-018-3955-8)
Supplement: Supplementary file 2 — Additional file 2: Figure S2. Complete sensorgrams for single-cycle kinetic analysis of VHHs binding to HER2 by SPR. [file 13104_2018_3955_MOESM2_ESM.pdf]

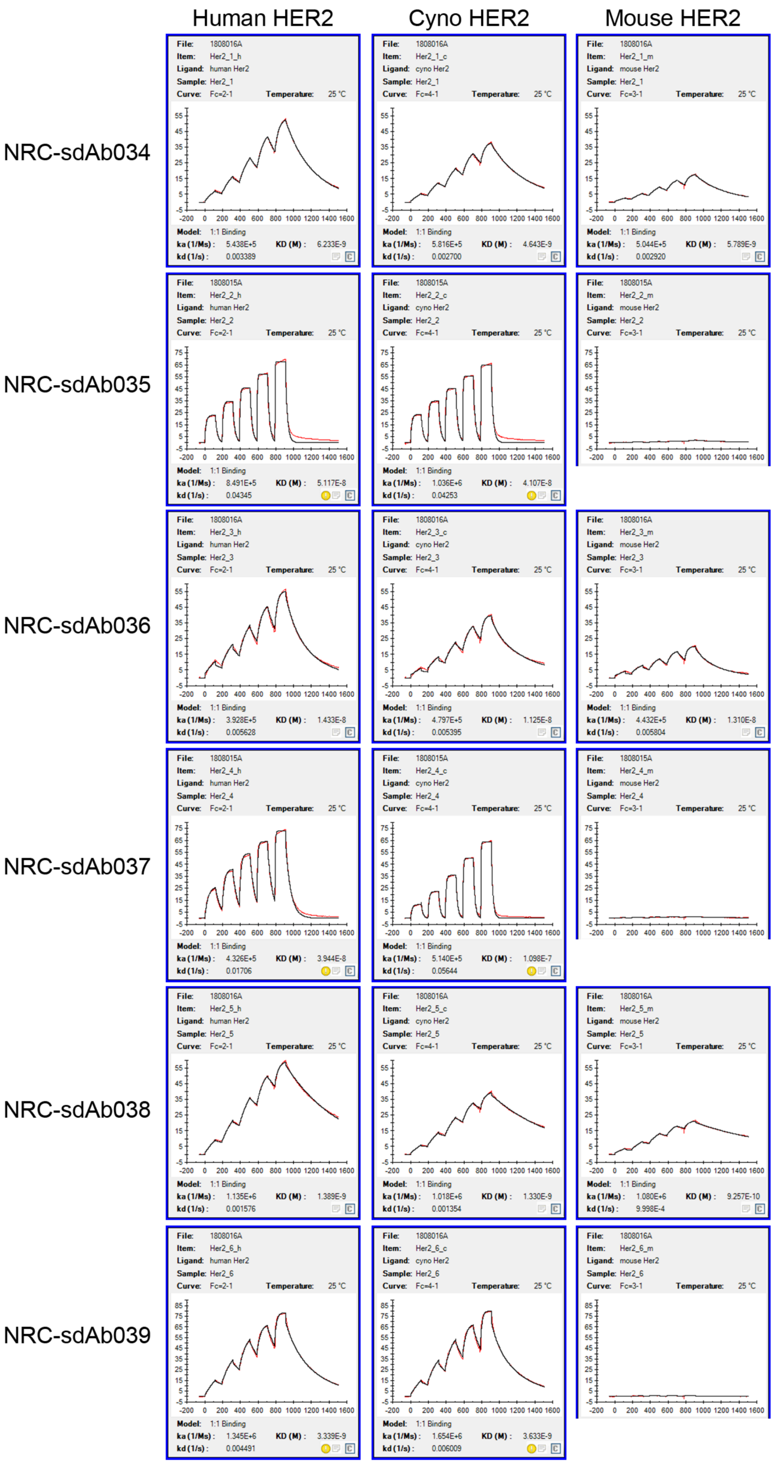

Figure S2. Complete sensorgrams for single-cycle kinetic analysis of V_HHs binding to HER2 by surface plasmon resonance.
