# Supplementary material for: Isolation and characterization of camelid single-domain antibodies against HER2
Source: BMC Res Notes. 2018 Dec 5;11:866. doi: 10.1186/s13104-018-3955-8 (PMC6282393; doi:10.1186/s13104-018-3955-8)
Supplement: Supplementary file 3 — Additional file 3: Figure S3. Complete sensorgrams for epitope binning SPR co-injection experiments. [file 13104_2018_3955_MOESM3_ESM.pdf]

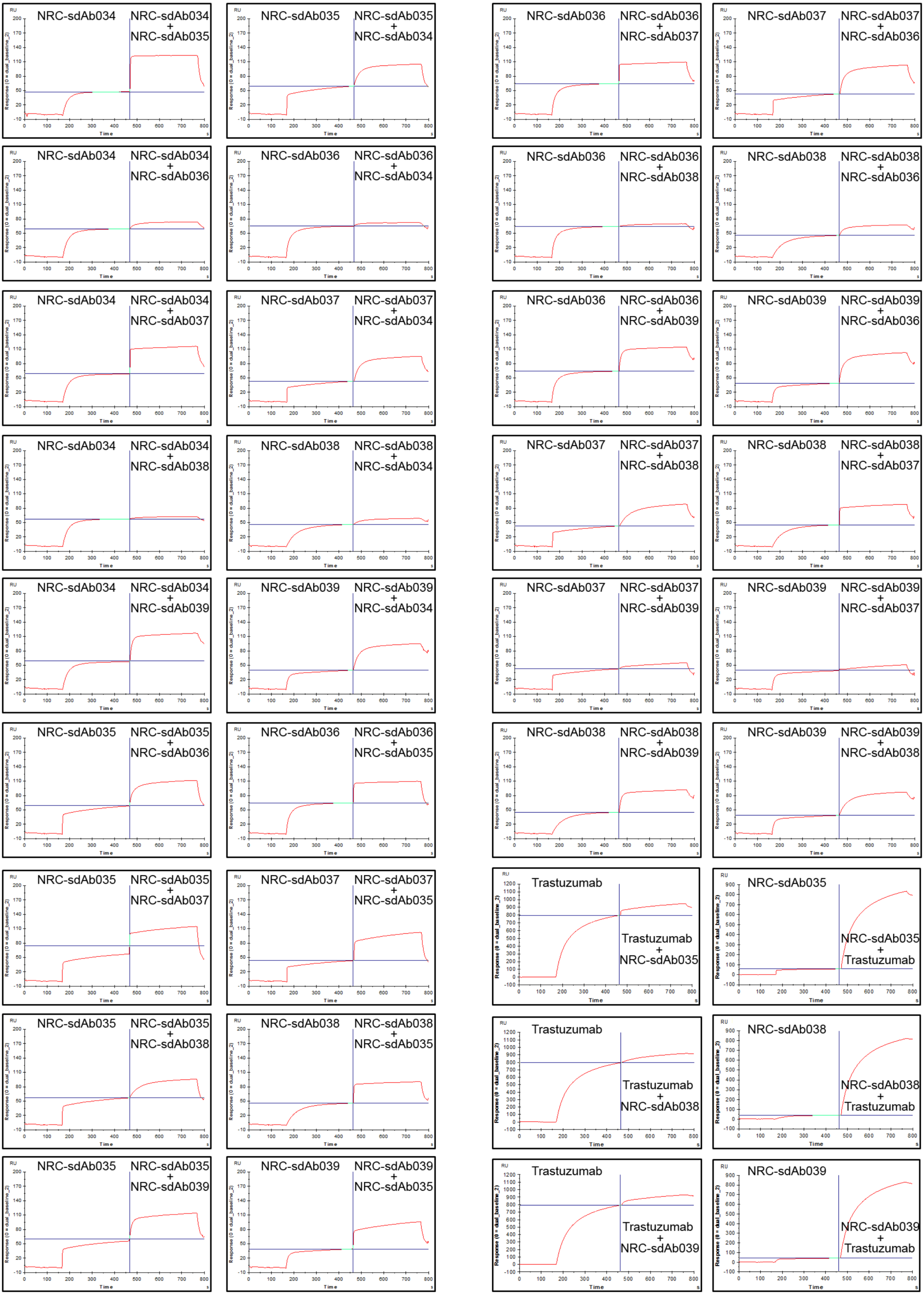

Figure S3. Complete sensorgrams for epitope binning SPR co-injection experiments. Data are shon for the human HER2 surface only. In the first injection, the HER2 surface was saturated using a concentration equivalent to 20× K_D of the first VHH (or 20 nM trastuzumab), then in the second injection, the first antibody was supplied in the presence of a second antibody (also 20× KD) to determine whether additional binding occurred. All possible permutations of antibodies were tested as described in the main text.
